# Consumption patterns of sweet drinks in a population of Australian children and adolescents (2003–2008)

**DOI:** 10.1186/1471-2458-12-771

**Published:** 2012-09-12

**Authors:** Britt W Jensen, Melanie Nichols, Steven Allender, Andrea de Silva-Sanigorski, Lynne Millar, Peter Kremer, Kathleen Lacy, Boyd Swinburn

**Affiliations:** 1 Research Unit for Dietary Studies, Institute of Preventive Medicine, Copenhagen Capital Region, Copenhagen University Hospital, Frederiksberg, Denmark; 2 Centre for Research in Childhood Health, Institute of Sports Science and Clinical Biomechanics, University of Southern Denmark, Odense, Denmark; 3 Centre for Intervention Research in Health Promotion and Disease Prevention (previous: Centre for Applied Research in Health Promotion and Prevention), The National Institute of Public Health, University of Southern Denmark, Copenhagen K, Denmark; 4 WHO Collaborating Centre for Obesity Prevention, Deakin University, Geelong, Australia; 5 Department of Public Health, University of Oxford, Oxford, United Kingdom; 6 Melbourne School of Population Health, University of Melbourne, Melbourne, Australia; 7 School of Psychology, Deakin University, Geelong, Australia

**Keywords:** Sweet beverages, Soft drink, Children and adolescents

## Abstract

**Background:**

Intake of sweet drinks has previously been associated with the development of overweight and obesity among children and adolescents. The present study aimed to assess the consumption pattern of sweet drinks in a population of children and adolescents in Victoria, Australia.

**Methods:**

Data on 1,604 children and adolescents (4–18 years) from the comparison groups of two quasi-experimental intervention studies from Victoria, Australia were analysed*.* Sweet drink consumption (soft drink and fruit juice/cordial) was assessed as one day’s intake and typical intake over the last week or month at two time points between 2003 and 2008 (mean time between measurement: 2.2 years).

**Results:**

Assessed using dietary recalls, more than 70% of the children and adolescents consumed sweet drinks, with no difference between age groups (p = 0.28). The median intake among consumers was 500 ml and almost a third consumed more than 750 ml per day. More children and adolescents consumed fruit juice/cordial (69%) than soft drink (33%) (p < 0.0001) and in larger volumes (median intake fruit juice/cordial: 500 ml and soft drink: 375 ml). Secular changes in sweet drink consumption were observed with a lower proportion of children and adolescents consuming sweet drinks at time 2 compared to time 1 (significant for age group 8 to <10 years, p = 0.001).

**Conclusion:**

The proportion of Australian children and adolescents from the state of Victoria consuming sweet drinks has been stable or decreasing, although a high proportion of this sample consumed sweet drinks, especially fruit juice/cordial at both time points.

## Background

Overweight and obesity is one of the most significant health burdens for the Australian population, and in 2007 23% of children aged 2 to 16 years were assessed as overweight or obese [1-3]. High intake of sweet beverages may be one causal factor in the prevalence of obesity [4]. DiMeglio and Mattes [5] suggest that this may be caused by limited or no compensation in total energy intake after ingestion of liquid carbohydrates, promoting an overall increased energy intake and positive energy balance. Soft drink and sugar-sweetened beverages have been associated with the development of overweight and obesity in childhood [6]. Several longitudinal studies [7-12] and a meta-analysis [13] have reported a direct association between intake of soft drink and other sweet drinks (a beverage category including fruit juice, fruit drink, cordial (a sweet, flavoured, concentrated syrup that is mixed with water to taste) and soft (carbonated) drinks) and weight gain in children and adolescents. Support for the association is not universal, however, with other longitudinal studies [14-17] and two meta-analyses [18,19] not supporting the association.

In Australia, as in other countries, beverage consumption patterns are likely to have changed in recent times; however we do not have a good understanding of the changes and their impact on child health outcomes. Further, little is known about how the patterns of sweet drink intake change in relation to demographics, gender and age. Recent Australian data on sweet drink consumption among children and adolescents are limited. A small number of state-based surveys [2,3,20] and two recent Australian studies have been published examining the beverage intake among Australian children and adolescents [21,22], however, these were all cross-sectional, and were therefore not able to assess the longitudinal changes in the beverage consumption. This is an important gap in explaining obesity patterns among young people, and a better understanding is required to facilitate targeted planning for future interventions. The aim of the present study was therefore, among children and adolescents in the state of Victoria, Australia, to:

1. Examine consumption patterns of sweet drinks (sugar-sweetened soft drink and fruit juice/cordial), and the relative contribution of each beverage type.

2. Determine if patterns of sweet drink consumption change as children age and if this is associated with gender.

3. Examine the secular changes in the patterns of consumption of sweet drinks.

## Methods

### Subjects

The analyses for this paper bring together the comparison group data (excluding the intervention group) where no intervention was carried out. The data were collected as part of the evaluation of two obesity prevention interventions; *Be Active Eat Well* (BAEW) and *It’s Your Move!* (IYM) (Table 1). The studies [23] were conducted in the Barwon-South Western Region of Victoria, Australia and included 5,224 children aged between 4 and 18 years. Data, including anthropometric measurements and dietary intake data, were collected at two time points between 2003 and 2008. The two studies are described briefly below and in more detail elsewhere [23,24]. 

**Table 1:** Overview of similarities and differences in the two studies BAEW and IYM

	**BAEW**	**IYM**
**Area**	Barwon-South Western Region of Victoria	Barwon-South Western Region of Victoria
**School level**	Primary school	Secondary school
**Age**	4-12 years	12-18 years
**Total n for study**	2,184 (845, from comparison group only, included in analysis)	3,040 (759, ,from comparison group only, included in analysis)
**Year of data collection**	Time 1: 2003/04, Time 2: 2006	Time 1: 2005, Time 2: 2008
**Mean duration between measurements**	2.1 years	2.3 years
**Intervention in comparison group**	None	None
**Beverages assessed**	Soft drink and fruit juice/cordial	Soft drink and fruit drink/cordial
**Dietary recall**	Yesterday (always a week day)	Last school day
**Frequency**	Last month	Last school week
**Respondent**	The child’s parent	The adolescent

BAEW, Be Active Eat Well.

IYM, It’s Your Move!

This study was conducted according to the guidelines laid down in the Declaration of Helsinki and all procedures involving human subjects were approved by Deakin University Human Research Ethics Committee (IYM: EC37-2004, BAEW: EC20-2003). Written informed consent was obtained from all participants and/or the parents of participants aged less than 18 years. In addition, verbal assent was obtained from all children and adolescents on the day of data collection.

### Be Active Eat Well (BAEW)

The evaluation of BAEW included 2,184 primary school children aged 4 to 12 years and their families. The intervention was conducted in the township of Colac, with a stratified random sample of the remainder of the Barwon-South Western Region of Victoria providing the comparison group (n = 1,183, 44% of the invited population) (Table 1) [24]. Baseline data were collected in 2003/04 (time 1) and post-intervention data in early/mid 2006 (time 2, mean duration between the measurements was 2.1 years) and included a computer assisted telephone interview (CATI) survey of the children’s parents with questions concerning the child’s food and beverage intake, physical activity and family socio-demographic characteristics [24,25]. From BAEW, 39 children aged between 12 and 13 years were excluded from this analysis to ensure that the age groups in the two studies did not overlap.

### It’s Your Move! (IYM)

IYM was one of four sites in the Pacific Obesity Prevention in Communities (OPIC) project [26] and included 3,040 adolescents aged between 12 and 18 years. The intervention was conducted in secondary schools in the East Geelong and Bellarine Peninsula regions of Victoria with a stratified random sample of schools from the Barwon-South Western Region of Victoria serving as the comparison group (n = 1,188, 47% of the invited population) (Table 1) [26]. The data were collected in winter/spring at two time points between 2005 and 2008 with a mean duration between measurements of 2.3 years. Data collection included an 83-question Adolescent Behaviours, Attitude and Knowledge Questionnaire completed by the adolescents [27]. This questionnaire was developed and used across the four sites of the OPIC study and included information on key behaviours such as dietary practices, physical activity and perceptions of school, home and neighbourhood environments.

Across the combined studies only children from the comparison groups with complete information on beverage intake at both time points were included in the analysis, resulting in 1,604 participants (845 from BAEW and 759 from IYM, 31% and 30% of the invited populations, respectively).

### Dietary assessment

One day’s intake of soft drink and fruit juice/cordial was assessed (along with other selected foods) using two questions at both time points as parent-report (BAEW) or self-report by the adolescent (IYM). In addition, intake of soft drink and fruit juice/cordial was reported per month (BAEW) or per week (IYM).

In BAEW the questions used to assess intake of soft drink and fruit juice/cordial at time 1 were:

*“How many cans of soft drink did the child drink yesterday? [By cans, we mean the 375 mls cans that you would see in supermarkets or convenience stores. Diet soft drinks are NOT included.]”* and *“How many serves of fruit juice or cordial did the child drink yesterday? [By serves we mean the small 250 ml tetra paks or a standard glass. The types of drinks include 100% fruit juices, diluted fruit juice drinks, and cordials]”.*

At time 2 the question assessing the intake of soft drink was slightly changed and referred to 250 ml glasses instead of 375 ml cans, while the question for intake of fruit juice/cordial remained unchanged. Furthermore, intake of soft drink and fruit juice/cordial was assessed as frequency per month in BAEW, with seven categorical ordinal response options ranging from less than once a month to two or more times per day. At both time 1 and 2 the intake of the participants in BAEW was assessed by the children’s parents.

In IYM the beverage intake was assessed in the Adolescent Behaviours, Attitude and Knowledge Questionnaire using the questions;

*“On the last school day, how many glasses or cans of non-diet soft drinks did you have?”* and, *“On the last school day, how many glasses of fruit drinks or cordial did you have?”.*

In IYM one glass/can of soft drink was assumed to equal 150/300 ml and one glass of fruit juice/cordial was assumed to equal 250 ml. In addition the frequency of soft drink and fruit juice/cordial intake during the last school week was assessed in IYM with possible responses ranging from none to five days.

The number of glasses/cans reported was converted to millilitres to enable comparison between the studies. The reported intakes of soft drink and fruit juice/cordial yesterday or last school day were used to derive total ‘sweet drinks’. ‘Diet’ and artificially sweetened beverages were not included in the assessment of any of the beverage categories.

### Socioeconomic status

Socioeconomic status (SES) was determined using the 2001 Socioeconomic Index for Areas (SEIFA) area level scores [28] based on the participant’s area of residence. The scores are based on a summary of several measures representing different aspects of relative socioeconomic advantage and disadvantage in a geographic area [23,24,28]. In this study, SEIFA values were dichotomised, dividing the areas into high/low SES based on the state wide median.

### Anthropometric measurements

Weight and height were measured at both time points in light clothing and without shoes by trained researchers according to standard methods for collecting anthropometric data in children [29].

Body mass index (BMI) was calculated from measured height and weight as kg/m^2^ and age- and gender- standardized BMI z-scores were calculated according to the WHO Child Growth Standard for children <5 years [30] and the Child Growth Reference for children >5 years [31]. Dichotomous weight status (healthy weight or overweight/obese) was defined according to the cut-points recommended by WHO. Due to the limited number of participants <5 years (n = 60) the cut point for children >5 years (overweight including obesity defined as a BMI z-score of >1 [32]) was used for all subjects including children <5 years in the present study.

### Statistical analysis

Descriptive statistics were calculated by age group and gender, and differences between these were tested by Chi-squared test for categorical data and ANOVA for continuous data. Spearman’s rank correlation coefficient was used to assess the correlation between yesterday’s intake (or last school day for IYM) and the intake last month (BAEW) or last school week (IYM) for soft drink and fruit juice/cordial for each study.

Beverage intake was reported as percentage of children who consumed the beverage on the previous day (BAEW) or previous school day (IYM) and percentage of children consuming ≤375 ml (one can), 376–749 ml (one to two cans) or ≥750 ml (more than two cans), respectively. Data are presented for all participants, by age group and gender, and as change in intake from time 1 to time 2. Differences between participants reporting increased, decreased or no change in intake from time 1 to time 2 were tested by Chi-squared test for categorical data and ANOVA for continuous data.

Difference in the proportion of consumers between time 1 and time 2 was tested by McNemar’s test for paired data in the longitudinal analysis. Secular changes in proportion of consumers, differences between gender and differences in proportion of consumers between age groups were tested by Chi-squared test. Secular changes in the proportion of participants consuming sweet drinks were assessed by comparing participants in each age group at time 1 with the participants who were at similar age at time 2. E.g. children aged 6 to <8 years in 2003/04 (time 1) were compared with those aged 6 to <8 years in 2006 (time 2, 4 to <6 years at time 1). A few participants only did not change age group from time 1 to time 2 and these were excluded from the analyses to ensure that the groups compared were independent.

Analyses were conducted using Stata version 11 (StataCorp LP, Texas, USA), and results were considered significant at p < 0.05. Bonferroni correction was used to take into account the multiple comparisons.

## Results

### Participants

Data on 1,604 children and adolescents were analysed for the current study. There were slightly fewer males than females in the sample (47.8%, no significant differences across age groups (NS)) and the mean age was 11.2 years (SD 3.7 years) (Table 2). BMI increased as expected with age (p < 0.0001) and overall the females had higher BMI than the males (p = 0.002). BMI z-score and weight status also differed by age (p < 0.0001 for both) but no significant difference was observed between genders (data not shown). The children and adolescents included in the study were generally from families residing in lower SES areas compared to the state wide median.

**Table 2:** Descriptive characteristics of participants at time 1

		**Age at time 1**							
		**BAEW**				**IYM**			
	**All**	**4 to < 6 years**	**6 to <8 years**	**8 to < 10 years**	**10 to <12 years**	**12 to <14 years**	**14 to <16 years**	**16 to <18 years**	**p‡**
**Distribution n (%)**	1604	169	214	278	184	309	277	173	
	(100.0)	(10.3)	(14.0)	(18.3)	(12.1)	(20.0)	(17.9)	(11.2)	
**Gender (males) n (%)**	766	87	97	144	87	152	132	67	0.16
	(47.8)	(51.5)	(45.3)	(51.8)	(47.3)	(49.2)	(47.7)	(38.7)	
**BMI**	19.6	16.7	16.8	18.2	19.2	21.0	21.6	22.7	<0.00001
	(3.6)	(1.8)	(2.1)	(2.8)	(3.0)	(3.8)	(3.2)	(3.3)	
BMI z-score mean (SD)*	0.6	0.9	0.6	0.9	0.7	0.6	0.4	0.4	<0.00001
	(1.0)	(1.0)	(1.0)	(1.0)	(1.0)	(1.1)	(0.9)	(0.9)	
**SES (below state wide median) n (%)§**	967	83	142	169	119	198	161	95	0.008
	(61.4)	(49.4)	(67.0)	(61.2)	(64.7)	(65.6)	(60.1)	(57.6)	
Overweight/ obese n (%)*	529	67	69	115	70	102	62	44	<0.0001
	(33.3)	(39.6)	(32.4)	(41.4)	(38.0)	(33.6)	(22.8)	(25.7)	

‡P-value for the difference between age groups.

*Due to missing data on weight and/or height, n is different for following groups; total n = 1591, 6 to <8 years n = 213, 12 to <14 years n = 304, 14 to <16 years n = 272, 16 to < 18 years n = 171.

§ Due to missing data on SES, n is different for following groups; total n = 1575, 4 to <6 years n = 168, 6 to <8 years n = 212, 8 to <10 years n = 276 12 to <14 years n=302, 14 to < 16 years n = 268, 16 to <18 years n = 165.

BAEW, Be Active Eat Well.

IYM, It’s Your Move!

BMI, body mass index.

BMI z-score, body mass index z-scores.

SES, socio-economic status.

Overview of distribution of age groups and descriptive characteristics of participants at time 1.

The correlation between one day’s intake and intake last week/ month ranged from 0.48 to 0.58 for soft drink (p < 0.00001) and 0.734 to 0.78 for fruit juice/cordial (p < 0.00001) differing by time point (time 1/time 2) and study (BAEW/IYM).

### Intake of sweet drinks

Overall 77% of the participants reported consuming any type of sweet drink (soft drink or fruit juice/cordial) yesterday or on the last school day (Table 3). The median intake of sweet drinks among consumers was 500 ml (data not shown), and almost a third (31%) of all children and adolescents consumed more than 750 ml (two cans or three glasses) (Table 4).

**Table 3:** Longitudinal changes in proportion of beverage consumers

			**Time 1**		**Time 2**		**p‡**
			**Consumers**		**Consumers**		
	**Age at time 1**	**Total n**	**n**	**(%)**	**n**	**(%)**	
**Sweet drinks (total)**	All	1604	1232	(76.8)	1145	(71.4)	<0.00001
	4 to <6 years	169	125	(74.0)	112	(66.3)	0.06
	6 to <8 years	214	157	(73.4)	140	(65.4)	0.04
	8 to <10 years	278	221	(79.5)	215	(77.3)	0.53
	10 to <12 years	184	146	(79.4)	141	(76.6)	0.54
	12 to <14 years	309	248	(80.3)	233	(75.4)	0.12
	14 to <16 years	277	206	(74.4)	188	(67.9)	0.06
	16 to <18 years	173	129	(74.6)	116	(67.1)	0.09
	p §			0.28		0.004	
**Fruit juice/cordial**	All	1604	1106	(69.0)	1017	(63.4)	0.0001
	4 to <6 years	169	111	(65.7)	102	(60.4)	0.26
	6 to <8 years	214	145	(67.8)	123	(57.5)	0.02
	8 to <10 years	278	199	(71.6)	192	(69.1)	0.49
	10 to <12 years	184	129	(70.1)	127	(69.0)	0.89
	12 to <14 years	309	222	(71.8)	201	(65.1)	0.05
	14 to <16 years	277	183	(66.1)	167	(60.3)	0.11
	16 to <18 years	173	117	(67.6)	105	(60.7)	0.13
	p §			0.62		0.06	
**Soft drink**	All	1604	532	(33.2)	442	(27.6)	0.0001
	4 to <6 years	169	36	(21.3)	23	(13.6)	0.04
	6 to <8 years	214	42	(19.6)	47	(22.0)	0.59
	8 to <10 years	278	80	(28.8)	69	(24.8)	0.27
	10 to <12 years	184	52	(28.3)	48	(26.1)	0.70
	12 to <14 years	309	153	(49.5)	124	(40.1)	0.009
	14 to <16 years	277	102	(36.8)	77	(27.8)	0.09
	16 to <18 years	173	67	(38.7)	54	(31.2)	0.12
	p §			<0.0001		<0.0001	

‡ P-value for the difference between the proportion of consumers between time 1 and time 2, tested by McNemar’s test.

§ P-value for the difference in the proportion of consumers between age groups, tested by Chi-squared test.

Intake of sweet drinks, fruit juice/cordial and soft drinks (yesterday or last school day) by baseline age groups at time 1 and time 2 presented as percentage consumers.

**Table 4:** Longitudinal changes in beverage consumption

	**Time 1**					**Time 2**			
		**None**	**≤375 ml**	**376-749 ml**	**≥750ml**	**None**	**≤375 ml**	**376-749 ml**	**≥750ml**
**Sweet drinks (total)**									
**All**	**n**	372	397	341	494	459	439	317	389
	**(%)**	(23.2)	(24.8)	(21.3)	(30.8)	(28.6)	(27.4)	(19.8)	(24.3)
**Males**	**n**	154	174	173	265	195	192	151	228
	**(%)**	(20.1)	(22.7)	(22.6)	(34.6)	(25.5)	(25.1)	(19.7)	(29.8)
**Females**	**n**	218	223	168	229	264	247	166	161
	**(%)**	(26.0)	(26.6)	(20.1)	(27.3)	(31.5)	(29.5)	(19.8)	(19.2)
p§		0.001					<0.0001		
**Fruit juice/cordial**									
**All**	**n**	498	441	323	342	587	477	268	272
	**(%)**	(31.1)	(27.5)	(20.1)	(21.3)	(36.6)	(29.7)	(16.7)	(17.0)
**Males**	**n**	216	206	166	178	258	219	140	149
	**(%)**	(28.2)	(26.9)	(21.7)	(23.2)	(33.7)	(28.6)	(18.3)	(19.5)
**Females**	**n**	282	235	157	164	329	258	128	123
	**(%)**	(33.7)	(28.0)	(18.7)	(19.6)	(39.3)	(30.8)	(15.3)	(14.7)
p§		0.04					0.009		
**Soft drink**									
**All**	**n**	1072	380	60	92	1162	306	96	40
	**(%)**	(66.8)	(23.7)	(3.7)	(5.7)	(72.4)	(19.1)	(6.0)	(2.5)
**Males**	**n**	492	179	37	58	507	169	61	29
	**(%)**	(64.2)	(23.4)	(4.8)	(7.6)	(66.2)	(22.1)	(8.0)	(3.8)
**Females**	**n**	580	201	23	34	655	137	35	11
	**(%)**	(69.2)	(24.0)	(2.7)	(4.1)	(78.2)	(16.4)	(4.2)	(1.3)
p§		0.002					<0.0001		

§ P-value for the difference in the distribution of consumer between genders, tested by Chi-squared test.

Intake of sweet drinks, fruit juice/cordial and soft drink by gender. Presented as proportion of consumers yesterday or last school day in four categories.

When the different types of sweet drinks were analysed separately, fruit juice/cordial was consumed by 69% of the children and adolescents (Table 3) with a median intake of 500 ml per day while 33% reported consuming soft drink (median intake 375 ml) (data not shown). More than one in five participants (21%) reported consuming more than 750 ml of fruit juice/cordial (Table 4). Among those who reported consuming soft drinks on the previous day, almost three quarters (71%) consumed the equivalent of one can of soft drink or less.

There were no significant differences in the proportion of participants consuming sweet drinks between the age groups at time 1 for all sweet drinks combined (p = 0.28) or fruit juice/cordial specifically (p = 0.62) (Table 3). However, significant differences between the age groups were found in the proportion of children and adolescents who reported consuming soft drink (p < 0.0001), with more consumers among the adolescents in secondary school compared to the children in primary school.

Significantly more males than females consumed any sweet drinks overall (p = 0.005) at time 1 and among older adolescents (age group 14 to <16 years p = 0.003, age group 16 to <18 years p = 0.01) (data not shown).

### Longitudinal changes over time

The longitudinal data showed that at time 1, 77% of the participants consumed any sweet drinks, while at time 2, the proportion was 71% (p < 0.00001) (Table 3). The prevalence of fruit juice/cordial consumption reduced from 69% at time 1 to 63% at time 2 (p=0.0001) and soft drink consumption prevalence was 33% at time 1 and 28% at time 2 (p=0.0001). This trend toward a decreasing proportion of consumers was observed in most age groups, but was only statistically significant in a few cases. After adjusting for multiple comparisons only the tests including all children and adolescents remained significant.

Almost half of the children in BAEW (44%) and just over one third of the adolescents in IYM (37%) reported constant intake of sweet drinks and another one third in both groups (BAEW: 33%, IYM: 38%) reported decreased intake from time 1 to time 2 while the remaining one quarter (BAEW: 23%, IYM: 25%) reported increased intake (Table 5). The consumption of fruit juice/cordial followed a similar pattern to overall consumption but the pattern of soft drink consumption differed. Compared to levels of consumption of fruit juice/cordial over time, a larger proportion of children and adolescents reported constant intake of soft drink and the majority of those were in the group that reported no consumption at both time points. A smaller proportion of children reported a change in levels of intake of soft drink.

**Table 5:** Persistence of beverage consumption patterns

		**Time 1**							
		**BAEW**				**IYM**			
**Sweet drinks (ml)**		**None n (%)**	**≤375 n (%)**	**376-749 n (%)**	**≥750 n (%)**	**None n (%)**	**≤375 n (%)**	**376-749 n (%)**	**≥750 n (%)**
**Time 2**	**None**	113	66	28	30	91	50	41	40
		(13.4)	(7.8)	(3.3)	(3.6)	(12.0)	(6.6)	(5.4)	(5.3)
	**≤375**	49	104	57	41	42	54	39	53
		(5.8)	(12.3)	(6.8)	(4.9)	(5.5)	(7.1)	(5.1)	(7.0)
	**376-749**	21	38	46	59	20	32	39	62
		(2.5)	(4.5)	(5.4)	(7.0)	(2.6)	(4.2)	(5.1)	(8.2)
	**≥750**	13	26	45	109	23	27	46	100
		(1.5)	(3.1)	(5.3)	(12.9)	(3.0)	(3.6)	(6.1)	(13.2)
**Fruit juice/cordial (ml)**									
**Time 2**	**None**	152	87	32	30	140	66	42	38
		(18.0)	(10.3)	(3.8)	(3.6)	(18.5)	(8.7)	(5.5)	(5.0)
	**≤375**	73	116	60	30	53	57	39	49
		(8.6)	(13.7)	(7.1)	(3.6)	(7.0)	(7.5)	(5.1)	(6.5)
	**376-749**	22	38	52	29	20	30	35	42
		(2.6)	(4.5)	(6.2)	(3.4)	(2.6)	(4.0)	(4.6)	(5.5)
	**≥750**	14	18	29	63	24	29	34	61
		(1.7)	(2.1)	(3.4)	(7.5)	(3.2)	(3.8)	(4.5)	(8.0)
**Soft drink (ml)**									
**Time 2**	**None**	531	92	3	32	335	136	24	9
		(62.8)	(10.9)	(0.4)	(3.8)	(44.1)	(17.9)	(3.2)	(1.2)
	**≤375**	67	20	2	14	83	86	20	14
		(7.9)	(2.4)	(0.2)	(1.7)	(10.9)	(11.3)	(2.6)	(1.8)
	**376-749**	27	20	0	15	9	14	9	2
		(3.2)	(2.4)	(0)	(1.8)	(1.2)	(1.8)	(1.2)	(0.3)
	**≥750**	10	8	0	4	10	4	2	2
		(1.2)	(1.0)	(0)	(0.5)	(1.3)	(0.5)	(0.3)	(0.3)

BAEW, Be Active Eat Well.

IYM, It’s Your Move!

The proportion of participants reporting increased, decreased or no change in intake of sweet drinks, fruit juice/cordial and soft drink from time 1 to time 2, based on the reported intake yesterday or last school day and presented by study.

The proportion of adolescents who reported increased, decreased or no change in intake of soft drink from time 1 to time 2 differed in gender (p < 0.001) and SES (p = 0.01). More males (23%) than females (10%) reported increased intake of sweet drinks, while more females (64%) than males (49%) reported no change in intake (data not shown). Also, more adolescents from families residing in low SES areas reported increased intake (low SES: 19%, high SES: 12%) while more adolescents from families residing in high SES areas reported decreased intake (low SES: 24%, high SES: 31%) from time 1 to time 2 and a similar trend was observed for sweet drink (p = 0.05) (data not shown). The proportion of children who reported increased, decreased or no change in intake of soft drink differed in SES (p < 0.001). More children from families residing in low SES areas reported decreased intake (low SES: 23%, high SES: 12%) while more children residing in high SES areas reported no change intake of soft drink (low SES: 60%, high SES: 73%) (data not shown). Furthermore, children who reported increased, decreased or no change in intake of sweet drinks and fruit juice/cordial differed in age (sweet drinks p = 0.004, fruit juice/cordial p = 0.02) with a trend toward that children who decreased their intake were younger, while those who increased their intake were older.

### Cross-sectional changes over time

The secular changes in sweet drink consumption were assessed using a serial cross-sectional analysis of the data in which the same age groups were compared at time 1 (BAEW: 2003/04, IYM: 2005) and time 2 (BAEW: 2006, IYM: 2008) (Table 6). A non-significant trend toward lower proportions of children and adolescents consuming any sweet drink at time 2 compared to time 1 was observed most age groups (Table 6). A significant difference was observed for age group 8 to <10 years where 80% of the children reported consuming sweet drinks at time 1 and 66% in the same age group at time 2 (p = 0.001). Overall a decreasing proportion of children and adolescents reported consuming any type of sweet drinks at time 2 compared to time 1.

**Table 6:** **Secular changes in proportion of beverage consumers**^**#**^

**BAEW**	**Time 1 (2003/4)**				**Time 2 (2006)**			**p§**
		**Consumers**			**Consumers**			
	**Age**	**n**	**(%)**	**Total n**	**n**	**(%)**	**Total n**	
**Sweet drinks (total)**	6 to<8 years	150	(73.9)	203	112	(66.3)	169	0.11
	8 to<10 years	218	(80.1)	272	134	(66.0)	203	0.001
	10 to <12 years	142	(79.9)	180	211	(77.6)	272	0.74
**Fruit juice/cordial**	6 to<8 years	138	(68.0)	203	102	(60.4)	169	0.13
	8 to <10 years	196	(72.1)	272	117	(57.6)	203	0.001
	10 to <12 years	125	(69.4)	180	188	(69.1)	272	0.94
**Soft drink**	6 to <8 years	42	(20.7)	203	23	(13.6)	169	0.73
	8 to <10 years	79	(29.0)	272	45	(22.2)	203	0.09
	10 to <12 years	51	(28.3)	180	69	(25.4)	272	0.49
**IYM**	**Time 1 (2005)**					**Time 2 (2008)**		**p§**
		**Consumers**			**Consumers**			
	**Age**	**n**	**(%)**	**Total n**	**n**	**(%)**	**Total n**	
**Sweet drinks (total)**	14 to <16 years	206	(74.4)	277	233	(75.4)	309	0.77
	16 to <18 years	75	(77.3)	97	188	(67.9)	277	0.08
**Fruit juice/cordial**	14 to <16 years	183	(66.1)	277	201	(65.1)	309	0.80
	16 to <18 years	68	(70.1)	97	167	(60.3)	277	0.09
**Soft drink**	14 to <16 years	102	(36.8)	277	124	(40.1)	309	0.41
	16 to <18 years	43	(44.3)	97	77	(27.8)	277	0.003

#Children and adolescents who remained in the same age group at both time points were excluded from these analyses.

§P-value for the difference between the proportion of consumers at time 1 compared to time 2, tested by Chi-squared test.

Intake of sweet drinks, soft drink and fruit juice/cordial by age and cohort, presented as percentage consumers yesterday or last school day.

When the two types of sweet drinks were analysed separately, the proportion of children and adolescents consuming fruit juice/cordial and soft drinks appeared to decrease from time 1 to time 2, however the difference was only significant in age group 8 to <10 years for fruit juice/cordial (p = 0.001) and 16 to <18 years for soft drink (p = 0.003) (Table 6). Adjusting for multiple comparisons the results remained significant.

## Discussion

This study examined the pattern of intake of sweet drinks, including nutritively sweetened soft drinks and fruit juice/cordial, among children and adolescents from Victoria, Australia. According to the dietary recalls more than 70% of the children and adolescents consumed sweet drinks and of these consumers, 40% had the equivalent of more than two cans (≥750 ml) of sweet drinks. Significantly more participants consumed fruit juice/cordial compared to soft drinks. The proportion of participants who reported consuming sweet drinks overall, and fruit juice/cordial specifically, varied with gender but remained stable across age groups, however there was a greater proportion of soft drink consumers in the older age groups. The proportion of children and adolescents consuming sweet drinks remained relatively stable over time, with some significant decreases in consumption, both longitudinally within cohorts, and in repeated cross-sections of comparable age groups.

### Intake of sweet drinks

The majority of existing research on sweet drink consumption has focused specifically on soft drinks. This study found that almost one third of the children and adolescents consumed soft drink -on the previous weekday. That is substantially higher than has been reported in other Australian studies. One study, from another Australian state (Queensland) found that between 11 and 19% of females and 12 to 28% of males had consumed soft drinks on the previous day, with a marked increase with age [20]. In contrast to the current study the Queensland sample did not include adolescents from age 16–18 years, where we found a high proportion of consumers. A more recent study, based on the 2007 Australian National Children’s Nutrition and Physical Activity Survey (NCNPAS), reported proportions of sweet drink consumers more similar to ours, in the range 19 and 38% among males and 17 and 29% among females for children aged 4–16 years [21]. The proportion of consumers of soft drink in the present study is; however, considerably lower than in other countries with comparable data. A 2004–05 study in Texas found that (depending on age) between 50 and 60% of children and adolescents aged 9–17 years consumed soft drink the previous day [33] and a Mexican study from 2006 reported that 76% of participants (5–11 years) consumed soft drink on a daily basis [34]. While there are methodological differences between these studies it seems likely that there are substantial differences in consumption of soft drink both between populations within Australia and between Australia and other countries. In addition, the prevalence of children and adolescents consuming any type of sweet drink (including both soft drink, sugar-sweetened beverages and fruit juice) is substantially higher in the United States (US) than was observed in the present study, according to analysis by Wang and colleagues based on the National Health and Nutrition Examination Surveys (NHANES) 1999–2004 [35]. They found that 90% of the participants (2–19 years) consumed any type of sweet drink the previous day [35] compared to 77% in the present study at time 1 and 71% at time 2.

In the present study more children and adolescents consumed fruit juice/cordial than soft drink, which is consistent with results from studies in the state of New South Wales [2,3]. In Texas, the proportion of children and adolescents consuming fruit juice, fruit-flavoured drinks and soft drinks was similar across all three categories of beverages (47-62%) [33]. The study based on the Australian NCNPAS reported that overall 37% of children and adolescents consumed fruit juice, 25% consumed soft drink and 20% consumed cordial on the previous day [21].

In the present study more participants in the older age groups consumed soft drinks. A similar trend has been observed in Queensland [20], nationally in the NCNPAS [21,22] and in the US [33]. In the present study there were no differences between the age groups in the proportion of children drinking fruit juice/cordial. It was observed that among the adolescents more males than females consumed any sweet drinks and also the volume of sweet drinks consumed was higher among males, consistent with previous findings from Australia [20-22], Mexico [34] and Texas, US [33].

### Longitudinal changes over time

In the cross-sectional analysis there was a higher proportion of consumers of soft drink in the older age groups. It was therefore expected that the proportion of consumers would increase with age (over time) in the longitudinal analysis. However, in the longitudinal analysis there was a non-significant trend towards a lower percentage of children and adolescents drinking any type of sweet drinks and fruit juice/cordial and soft drinks separately after approximately two year follow-up, although this was only significant for some age groups.

### Cross-sectional changes over time

In the analysis of secular changes in consumption from time 1 to time 2 a similar non-significant trend was seen. These findings indicate a stable or even decreasing percentage of Victorian children drinking any type of sweet drink. Even though no intervention was made in the comparison groups the inclusion of the children and adolescents in the studies may have lead to greater awareness on healthy eating in general and decreased intake of sweet drinks and/ or decreased reported intake of sweet drinks. Furthermore, in BAEW the data at time 1 were collected over a long time period including summer/autumn while data at time 2 were collected in winter/spring, which may have affected the results as well. A recent study from Queensland, however, reported similar results as found in the present study for soft drink and concluded that there was a reduction of soft drink consumption in 2008 compared to 2003 [36]. Preliminary analysis from NHANES in the US (2003–04 and 2005–06) also indicate a decrease in the average intake of soft drink and fruit juice among US children [37]. The Australian Beverage Industry has reported a decrease in sales of sugar-sweetened carbonated soft drinks and an increase in sales of non-sugar-sweetened carbonated soft drink and water between 1997 and 2006, and especially from 2002 [38] which supports the observed trends in the present data.

The results from the present study also indicate a lower volume of soft drink intake at time 2 among the children (4–12 years) compared to time 1, however, the question assessing soft drink intake in BAEW was slightly altered from time 1 to time 2 and this may have had some effect on the apparent changes in intake of soft drink in most of the age groups from BAEW. Based on the data presented by the Australian Beverage Industry reporting an increase in the sale of diet beverages and water among the adult population [38] it is possible that some children and adolescents also have changed the intake of regular (sugar-sweetened) soft drink to diet soft drink or packaged water. In addition, there were substantial changes over the study period in the regulation and operation of school canteen services in Victoria, which may have impacted on the availability of sweet drinks on school days [39,40].

### Strengths

The strengths of the study include the collection of data on both one day’s intake (yesterday/ last school day) and intake last week or month. The correlation between the two measurements is moderate to high [41], which supports the use of the single day recall questions that are generally simpler for participants to answer. The longitudinal design provides the opportunity to study changes in beverage intake within the same group of children over time and the combination of two closely related studies enabled assessment of beverage consumption patterns across childhood and adolescence in a large sample. The two studies had very similar designs, and methodological differences between them were relatively minor. One exception to this was the dietary data being self-reported by adolescents (IYM) and parent-reported for primary school children (BAEW); however, given the ages of the participants, these methods are most appropriate for obtaining accurate recall data. Furthermore, the use of comparison groups meant that only the background health promotion activities were operating in the populations.

### Limitations

The limitations of the study include that the analyses were based on single 24-hour reported beverage consumption, either yesterday’s intake or intake on the last school day, which may not reflect long term intake due to day-to-day variation in food and beverage consumption. Furthermore, the use of one day’s intake as an indicator of sweet drink consumption may increase the risk of misclassification and thereby the probability of type II errors. Moreover, the reported beverage intake is potentially subject to the limitations inherent in most dietary assessment methods including recall bias [42], underreporting [43] and social desirability bias [44], which may be especially pronounced for snack and sugar intakes [42]. Additionally, parents may not be fully aware of what their child drinks during the day, especially when the child is away from home [42]. In both studies only intakes of selected foods and beverages were assessed which imposes a substantially lower burden on the respondents [43] and may result in higher participation rates, among the adolescents compared to a 24-hour recall; however it precludes analyses that consider total energy or other nutrient intakes.

Due to the geographic limitation of the study, the results may not be generalizable to all Australian children and adolescents. Also the large number of participants lost to follow-up and the over representation of families residing in low SES areas restrict the generalisability of the results of the study. Generally, children and adolescents lost to follow-up between time 1 and time 2 had a higher intake of both soft drink and fruit juice/cordial (BAEW: soft drink p = 0.004, fruit juice/cordial p = 0.03, IYM: soft drink p < 0.0001, fruit juice/cordial p = 0.08) than those participating at both time points. No difference was observed in BMI between children who participated at both time 1 and time 2 and children who were lost to follow-up after time 1 (p = 0.37), while the adolescents who were lost to follow-up in IYM tended to have higher BMI than adolescents who remained in the study (p = 0.06).

Beverage intake was reported only for weekdays, which may not represent usual intake, since it has been demonstrated that children eat differently on weekend-days compared to school days [45] and have a higher intake of soft drinks, but not fruit juice/cordial in the weekends [46]. Thus, the intake of sweet drinks reported in the present study may be lower than the actual intake. Although other studies have found that the majority of sugar-sweetened beverages was consumed at home [21,22] the children and adolescents reported a high intake of sweet drinks in the present study, and at least some of this was likely to be consumed in school. Moreover, the intake of fruit juice/cordial was assessed in a combined question and it was therefore not possible to get separate information on these two beverages. Furthermore, no information was obtained on intake of other beverages such as diet beverages, milk and water and we are therefore not able to determine whether the decreased intake of sweet drinks is associated with increased intake of other types of beverages.

A final limitation of the studies is that the questionnaires not have been formally validated in their current form. The questions were selected on the balance of validation evidence from current literature and the feasibility of measuring large number of children. Moreover, the majority of the questions was taken or adapted from previously validated and widely used surveys in Australia and New Zealand and pilot testing was performed with both questionnaires before their use in the program evaluations [27].

## Conclusion

This study showed that a large proportion of children and adolescents consumed sweet drinks, and of those, a higher proportion reported consuming fruit juice/cordial compared to soft drinks. The proportion of participants who reported consuming sweet drinks overall, and fruit juice/cordial specifically remained stable across age groups while there were a greater proportion of soft drink consumers in the older age groups. Overall, the proportion of consumers and the consumption of sweet drinks were high in this population and remained relatively stable over time. Although this study showed some evidence of a decrease in the intake from 2003 to 2008, there is still a great deal of room for improvement in the beverage intake of this population of Australian children and adolescents.

## Abbreviations

BAEW: Be Active Eat Well; IYM: It’s Your Move!; CATI: computer assisted telephone interview; OPIC: Pacific Obesity Prevention in Communities; BMI: body mass index; BMI z-score: body mass index z-score; US: United States; NHANES: the National Health and Nutrition Examination Survey; NCNPAS: National Children’s Nutrition and Physical Activity Survey; SES: socioeconomic status; SEIFA: Socioeconomic Index for Areas.

## Competing interests

The authors have no conflicts of interest to declare.

## Author’s contribution

AdSS, PK, BS and LM and were involved in the design, conduct and/or analysis of the intervention study evaluations. MN, SA and BWJ conceived the idea for this manuscript. BWJ conducted the data analyses and drafted the manuscript with supervision from SA and MN. All authors reviewed drafts of the manuscript and approved the final paper as submitted.

BWJ was supported by the TrygFoundation, Centre for Intervention Research in Health Promotion and Disease Prevention (previous: Centre for Applied Research in Health Promotion and Prevention), The Danish Heart Foundation (10-04-R79-A2844-22578), the “Familien Hede Nielsen” foundation and University of Southern Denmark.

AdSS is supported by an NHMRC Capacity Building Grant and the Jack Brockhoff Foundation.

## Pre-publication history

The pre-publication history for this paper can be accessed here:

http://www.biomedcentral.com/1471-2458/12/771/prepub
